# Fluoroquinolone-resistant *Escherichia coli*, Indonesia

**DOI:** 10.3201/eid1109.041207

**Published:** 2005-09

**Authors:** Kuntaman Kuntaman, Endang Sri Lestari, Juliëtte A. Severin, Irma M. Kershof, Ni Made Mertaniasih, Marijam Purwanta, Usman Hadi, James R. Johnson, Alex van Belkum, Henri A. Verbrugh, on behalf of the Antimicrobial Resistance in Indonesia, Prevalence and Prevention study group

**Affiliations:** * Airlangga University, Surabaya, Indonesia;; † Diponegoro University, Semarang, Indonesia;; ‡ Erasmus Medical Center, Rotterdam, the Netherlands;; § Minneapolis Veterans Affairs Medical Center, Minneapolis, Minnesota, USA

**Keywords:** Escherichia coli, Indonesia, fluoroquinolones, drug resistance, research

## Abstract

In a recent, population-based survey of 3,996 persons in Indonesia, fluoroquinolone (FQ)-resistant *Escherichia coli* was prevalent in the fecal flora of 6% of patients at hospital admission and 23% of patients at discharge, but not among healthy relatives or patients visiting primary healthcare centers (2%). Molecular typing showed extensive genetic diversity with only limited clonality among isolates. This finding suggests that independent selection of resistant mutants occurs frequently. FQ-resistant isolates exhibited a higher rate of spontaneous mutation, but sparser virulence profiles, than FQ-susceptible isolates from the same population. The resistant isolates belonged predominantly to phylogenetic groups A (57%) and B1 (22%) but also to the moderately virulent group D (20%). Hypervirulent strains from the B2 cluster were underrepresented (1%). Because FQ-resistant *E. coli* can cause disease, especially nosocomial infections in immunocompromised patients, spread of such strains must be stopped.

## Abstract 2

High prevalence may be due to clonal spread and emergence of resistant strains.

*Escherichia coli* is a common constituent of the gastrointestinal flora of most vertebrates, including humans, and may be isolated from a variety of environmental sources. While most strains are nonpathogenic, certain ones can cause a variety of intestinal and extraintestinal infections. Pathogenicity is largely determined by gene-encoding virulence factors (VFs), such as adhesins, toxins, and polysaccharide surface coatings (*1*). Phylogenetic analysis showed that most *E. coli* strains fall into 4 main phylogenetic groups, designated A, B1, B2, and D (*2*). *E. coli* strains that cause extraintestinal infections derive predominantly from group B2 and, to a lesser extent, group D. Strains of groups A and B1 represent most commensal strains and are largely devoid of virulence determinants (*3*). Although strains harboring a robust extraintestinal VF repertoire cluster predominantly in groups B2 and D, isolates within each phylogenetic group can be further classified as extraintestinal pathogenic *E. coli* (ExPEC) or non-ExPEC depending on whether specific virulence traits are present (*4**,**5*).

The fluoroquinolones (FQs) are potent antimicrobial agents used for the treatment and prophylaxis of infections caused by gram-negative bacteria, including *E. coli*. FQ-resistant *E. coli* has been reported increasingly during the last decade in both the hospital environment and the community, which may ultimately limit the utility of these broad-spectrum agents (*6**–**8*). Moreover, FQ-resistant *E. coli* strains often show resistance to other drugs, such as ampicillin, tetracycline, chloramphenicol, trimethoprim-sulfamethoxazole, and gentamicin (*7**,**9*). Recent reports have suggested that clinical FQ-resistant *E. coli* actually tends to be less virulent than susceptible isolates. FQ-resistant *E. coli* from hospitalized Dutch patients derived predominantly from the low-virulence phylogenetic groups A and B1. None of the 13 invasive isolates derived from phylogenetic group B2 (*10*). In addition, evidence suggests that clinical FQ-resistant *E. coli* isolates from humans in Iowa were associated with a shift toward non-B2 phylogenetic groups and to a lower overall virulence genotype (*4*). FQ resistance may also be associated with strains that intrinsically have a higher overall mutation rate, since the resistance to FQs in *E. coli* involves the accumulation of multiple spontaneously occurring point mutations in several genes (*9**,**11*). These associations, however, may depend on the strains' geographic or clinical origin.

In our study, we investigated these putative associations in a well-defined collection of isolates from Indonesia. A population-based survey of ≈4,000 people in 2 cities on the island of Java (Surabaya and Semarang) was initiated in 2000 by the Antimicrobial Resistance in Indonesia, Prevalence and Prevention study group to investigate the level of carriage of resistant microorganisms. FQ-resistant *E. coli* was prevalent in the fecal flora of 6% of patients at hospital admission and 23% of patients at discharge but not among healthy relatives or patients visiting primary healthcare centers (2% in both groups) (ES Lestari, unpub. data). In our study, we analyzed these FQ-resistant *E. coli* isolates to elucidate their molecular epidemiology and virulence. To define clonal relatedness, we performed enterobacterial repetitive intergenic consensus (ERIC) polymerase chain reactions (PCR). The phylogenetic background and virulence profile of these isolates were determined by PCR methods and compared with similar data for FQ-susceptible *E. coli* isolated from the same population. Finally, we examined the link between FQ resistance and the intrinsic mutation rate.

## Materials and Methods

### Strains

The study group program surveillance was initiated to determine the prevalence of antimicrobial resistance in Indonesia. Four different groups of persons in Surabaya and Semarang were studied for carriage of resistant microorganisms in their stools. The 4 groups were patients on the day of admission to the hospital (group 1), patients on the day of discharge after >5 days of hospitalization (group 2), patients visiting a primary healthcare center (group 3), and healthy relatives or household members of group 1 patients (group 4). In groups 1 and 2, rectal swabs were taken from patients in the internal medicine, surgery, gynecology/obstetrics, or pediatrics departments. The specimens were collected from July to October 2001 in Surabaya and from January to May 2002 in Semarang. Further details on the methods of culturing will be published elsewhere. A total of 5,535 *E. coli* isolates from 3,284 patients were cultured. Antimicrobial susceptibility testing was performed for 1 isolate per patient. The overall by-isolate prevalence of resistance to ciprofloxacin as determined by disk diffusion was 8%. The prevalence of resistance was highest among patients on the day of discharge (18% in Surabaya and 27% in Semarang) and lowest among patients visiting primary healthcare centers and among family members of patients admitted to the hospital (2% in both groups). The prevalence of FQ-resistant *E. coli* among patients who were tested on the day of admission was 8% in Surabaya and 4% in Semarang. We studied 196 FQ-resistant isolates in more detail. Seventy-five (38%) of these were from Surabaya (19, 48, 4, and 4 isolates from stated population groups 1, 2, 3, and 4, respectively) and 121 (62%) from Semarang (13, 92, 11, and 5, respectively). The FQ-resistant isolates were recovered from patients from all 4 hospital departments in both cities. In Semarang, 43% of these isolates were from surgery departments and 41% were from internal medicine departments. In Surabaya, 43% of the isolates were from the internal medicine department. All 196 ciprofloxacin-resistant *E. coli* and 200 ciprofloxacin-susceptible *E. coli* (20 randomly chosen isolates from groups 1, 2, and 3 and 40 from group 4, from each city) were confirmed by Vitek 2 (bioMérieux, Marcy-l'Etoile, France) according to the manufacturer's instructions and included in the molecular analyses.

### DNA Isolation

Bacterial DNA was isolated by using the MagNA Pure LC with the MagNA Pure LC DNA Isolation Kit III for bacteria and fungi (standard protocol; Roche Molecular Biochemicals, Mannheim, Germany). DNA concentration was assessed spectophotometrically. Samples were frozen at –20°C until used.

### Bacterial Typing by ERIC-PCR

ERIC-PCR was conducted with primers ERIC-1R and ERIC-2 as described previously (*8**,**12**,**13*). The amplification products were subjected to electrophoresis in a 1% agarose gel and were stained with ethidium bromide (50 μg/mL). The ERIC-PCRs were performed by 1 technician within 1 month. Profiles were visually analyzed by 2 microbiologists. Single-band differences in profiles among strains led to the definition of separate genotypes. Ambiguous isolates were retested and analyzed by 2 other microbiologists.

### Phylogenetic Analysis and Virulence Typing

Isolates were assigned to 1 of 4 main *E. coli* phylogenetic groups (A, B1, B2, and D) according to an established triplex PCR assay, in which the 4 phylogenetic groups yield distinct combinations of 3 possible PCR products, *chuA* (heme transport), *yjaA* (unknown function gene from *E. coli* K-12 genome), and TSPE4.C2 (anonymous fragment identified by subtractive hybridization) (*2**,**14*). All isolates were screened for 5 ExPEC-defining virulence markers, *papA*/*papC*, *sfa*/*focDE*, *afa*/*draBC*, *kpsM* II, and *iutA*. Based on previous statistical analyses of similar data, from collections within which each isolate's ExPEC status could be inferred based on ecologic source or experimental virulence, isolates were classified as ExPEC if positive for >2 of these 5 defining virulence markers (*4*). All isolates were also screened for *hlyD* (hemolysin), another ExPEC-associated VF. Subsequently, all isolates that satisfied molecular criteria for ExPEC were screened for 32 additional virulence markers^3^. These virulence genes were detected by a combination of multiplex PCR and dot-blot hybridization with primers specific for internal or flanking sequences and probes generated and labeled with these primers; this method was previously validated by using dot-blot hybridization with defined control strains (*15*). A VF score was calculated for each strain as the sum of all VF genes for which the strain tested positive. In all of these PCR assays, the identity of the PCR products was deduced by comparing their size to molecular size standards in ethidium bromide–stained agarose gels. Appropriate positive and negative controls were included in each run.

### Mutation Rate Analysis

The mutation rate was determined for 20 randomly selected isolates from phylogenetic group A (10 FQ-susceptible and 10 FQ-resistant) by monitoring the isolates' capacity to generate mutations conferring resistance to rifampin, as described previously (*9**,**16*). Forty independent cultures of each of the 20 strains were set up in Luria broth. After overnight incubation, equal concentrations of cultures were suspended in 0.85% NaCl. The suspensions were spread on Luria agar plates containing 100 μg/mL rifampin and incubated overnight. For each strain, the proportion of cultures giving no resistant mutants was used to calculate the mutation rate per cell per generation according to the fluctuation test of Luria and Delbrück. To avoid confounding by variation in phylogenetic background, only phylogenetic group A isolates were investigated. For comparisons of results, we used the relative mutation rate, which was defined as the rate relative to the rate for *E. coli* strain Nu14 (5 × 10^–9^ per cell per generation) (*9*).

### Statistical Analysis

All data were analyzed by using the statistic software packages SPSS version 10.0 (SPSS, Chicago, IL, USA) and EpiInfo version 5.00 (Centers for Disease Control and Prevention, Atlanta, GA, USA). Chi-square or Fisher exact tests (2-tailed) were used when appropriate for comparisons of proportions. Comparisons involving VF scores and relative mutation rates were analyzed by using the Mann-Whitney *U* test. The criterion for statistical significance was a p value <0.05.

## Results

### Spread of FQ-resistant *E. coli*

Genetic heterogeneity among the 196 FQ-resistant *E. coli* was assessed by ERIC-PCR. We documented 158 different patterns, designated types 1–158, which indicated a genetically diverse collection of strains. Twenty pairs of isolates with identical profiles were identified, and 9 distinct multiple-isolate clones were represented by isolates from 3 patients each. The limited number of shared genotypes was mainly recovered from group 2 patients, i.e., patients at the time of discharge from the hospital, 49 (73%) of 67 isolates. Among the total number of 140 isolates from group 2, we identified 119 different ERIC-PCR profiles.

Type 37 occurred in 3 patients from the internal medicine department in Surabaya; all 3 patients were present within this department on the same day. The finding of this unique isolated cluster can be explained by patient-to-patient transmission or a nonpatient-associated environmental source. This explanation was not further examined in this study. Type 90 was isolated from 2 patients on the day they were discharged from the internal medicine department in Semarang. Samples were collected on consecutive days. An isolate with an identical ERIC-PCR pattern was found in the same period in the same hospital in a pediatric patient at discharge. No further obvious clustering in time and place was observed among isolates from the 9 multiple-strain clusters.

### Phylogenetic Analysis

PCR-based phylotyping showed that the 200 FQ-susceptible isolates were predominantly from phylogenetic groups A (52%) and B1 (30%) (Table 1). The 196 FQ-resistant isolates also mainly derived from phylogenetic groups A (57%) and B1 (22%), but some derived from the moderately virulent phylogenetic group D (20%). Hypervirulent strains from the B2 cluster were underrepresented (1%). Eighteen (67%) of the 27 isolates from the 9 distinct clones that were represented by 3 isolates each belonged to group A.

**Table 1:** Distribution of phylogenetic groups and virulence factors*

Group or factor	All isolates (n = 396)	FQ-susceptible (n = 200)	FQ-resistant (n = 196)	p values
A	215 (54)	103 (52)	112 (57)	NS
B1	102 (26)	59 (30)	43 (22)	NS
B2	17 (4)	15 (8)	2 (1)	0.001
D	62 (16)	23 (12)	39 (20)	0.02
*papA*	28 (7)	27 (14)	1 (1)	<0.001
*papC*	29 (7)	28 (14)	1 (1)	<0.001
*sfa*/*focDE*	8 (2)	8 (4)	0	0.007
*afa*/*draBC*	11 (3)	11 (6)	0	0.001
*iutA*	131 (33)	61 (31)	70 (36)	NS
*hlyD*	20 (5)	20 (10)	0	<0.001
*kpsM* II	56 (14)	54 (27)	2 (1)	<0.001
ExPEC	44 (11)	40 (20)	4 (2)	<0.001

*FQ, fluoroquinolone; ExPEC, extraintestinal pathogenic *Escherichia coli*; NS, not significant. Screening for ExPEC was performed for 199 FQ-susceptible and 195 FQ-resistant isolates. Data are no. (%) of isolates.

Table 1 shows that the resistant isolates were significantly depleted for phylogenetic group B2 and enriched for group D, when compared with the susceptible isolates. These shifts in phylogenetic distribution were significant both overall and specifically in Semarang, whereas a similar but nonsignificant trend was observed in Surabaya.

The phylogenetic distribution of all 396 isolates among the 2 cities was highly similar (data not shown). Comparisons of the distributions among the 4 population groups showed that group D isolates were more often obtained from patients sampled on the day of discharge than from other population groups (37 [21%] of the 180 group 2 isolates belonged to group D versus 25 [12%] of the 216 nongroup 2 isolates, p = 0.01). Stratification showed, however, that this association was due to the excess prevalence of FQ-resistant group D isolates among the group 2 patients. Furthermore, B2 isolates were significantly more prevalent in group 3, patients visiting public healthcare centers (7 [13%] of the 55 group 3 isolates belonged to group B2 versus 10 [3%] of the 341 nongroup 3 isolates, p = 0.004).

### Virulence Typing

All *E. coli* isolates were tested for a set of virulence factors to allow an inference as to their pathogenic potential. The overall prevalence of the 5 defining ExPEC VFs ranged from 2% (*sfa*/*focDE*) to 33% (*iutA*) (Table 1). The FQ-resistant isolates were significantly depleted for *papA*, *papC*, *sfa*/*focDE*, *afa*/*draBC*, *hlyD*, and *kpsM II* (Table 1), when compared with the susceptible isolates. Accordingly, 40 (20%) FQ-susceptible *E. coli* isolates, but only 4 (2%) FQ-resistant isolates (2%), were classified as ExPEC, as they exhibited >2 of the 5 key ExPEC VFs (p<0.001). Thus, FQ resistance was associated with reduced inferred virulence. All FQ-resistant *E. coli* isolates from the 9 distinct clones that were represented by 3 patients each were found to be non-ExPEC.

The distribution of the 6 screening VFs was also analyzed in relation to the 4 phylogenetic groups (Table 2). Each VF was broadly distributed, occurring in >3 phylogenetic groups. However, *papA*, *papC*, *kpsM II*, *hlyD*, and *sfa*/*focDE* were all significantly associated with phylogenetic group B2. Accordingly, 53% of the phylogenetic group B2 isolates qualified as ExPEC versus 9% of the non-B2 isolates (p<0.001) (Tables 2 and 3).

**Table 2:** Distribution of virulence factors

Virulence factor	All isolates (n = 394)	Phylogenetic group, n (%)			
		A (n = 215)	B1 (n = 101)	B2 (n = 17)	D (n = 61)
*papA*	28 (7)	7 (3)	11 (11)	8 (47)*	2 (3)
*papC*	29 (7)	8 (4)	11 (11)	8 (47)*	2 (3)
*iutA*	131 (33)	66 (31)	32 (32)	8 (47)	25 (41)
*kpsM* II	56 (14)	19 (9)	23 (23)	11 (65)*	3 (5)
*hlyD*	20 (5)	8 (4)	7 (7)	5 (29)*	0
*sfa*/*focDE*	8 (2)	2 (1)	4 (4)	2 (12)*	0
*afa*/*draBC*	11 (3)	6 (3)	2 (2)	1 (6)	2 (3)

*p<0.05.

**Table 3:** Distribution of phylogenetic groups and virulence factors*

Characteristic	Prevalence of associated characteristic, n (%)			
	All isolates (n = 44)	FQ-susceptible (n = 40)	FQ-resistant (n = 4)	p values
Group A	16 (36)	14 (35)	2 (50)	NS
Group B1	14 (32)	14 (35)	0	NS
Group B2	9 (21)	9 (23)	0	NS
Group D	5 (11)	3 (8)	2 (50)	NS
Surabaya	23 (52)	22 (55)	1 (25)	NS
Semarang	21 (48)	18 (45)	3 (75)	NS
Admission	13 (30)	12 (30)	1 (25)	NS
Discharge	16 (36)	14 (35)	2 (50)	NS
PHC	10 (23)	9 (23)	1 (25)	NS
Relatives	5 (11)	5 (13)	0	NS
*iha*	25 (58)	25 (64)	0	0.025
*sat*	25 (58)	25 (64)	0	0.025
*fyuA*	35 (81)	35 (90)	0	0.001
*ibeA*	3 (7)	1 (3)	2 (50)	0.019
*malX*	26 (60)	26 (67)	0	0.019

*FQ, fluoroquinolone; NS, not significant; PHC, primary healthcare center. Extended virulence typing was performed for 39 FQ-susceptible isolates and 4 FQ-resistant isolates. Only those virulence factors are shown for which the comparison of FQ-resistant extraintestinal pathogenic *Escherichia coli* (ExPEC) to FQ-susceptible ExPEC was significant.

The 44 ExPEC isolates were studied in more detail (Table 3). The ExPEC isolates derived mainly from phylogenetic groups A (36%) and B1 (32%), with the 4 FQ-resistant ExPEC isolates belonging to groups A (n = 2) and D (n = 2). Many (36%) ExPEC isolates originated from patients on the day of discharge. Both of the FQ-resistant ExPEC isolates from group 2 were from patients in the surgical ward in Semarang. Again, no evidence for clonality was seen. The 4 resistant ExPEC isolates exhibited sparse VF profiles when compared with the susceptible ExPEC isolates. These isolates lacked classic ExPEC VFs such as *focG*, *hlyD*, and *cnf1*. Four other VFs, *iha*, *sat*, *fyuA*, and *malX*, were more prevalent among susceptible, rather than resistant, ExPEC isolates. Only *ibeA* was more prevalent among the resistant isolates. The VF *iutA* was detected in all FQ-resistant ExPEC isolates and in 27 (68%) of the 40 FQ-sensitive isolates. This difference was not significant. Aggregate VF scores were lower among FQ-resistant ExPEC isolates (median 6, range 4–8) than among the 40 FQ-susceptible ExPEC isolates (median 10, range 3–16, p = 0.024).

### Mutation Rate

The link between mutation rate and resistance to FQs was studied, as the rate of mutation accumulation might be a factor in the development of FQ resistance. The 10 FQ-susceptible isolates had relative mutation rates of <0.52 (median rate 0.32, range 0.03–0.52), whereas the 10 FQ-resistant *E. coli* exhibited relative mutation rates of >0.55 (median rate 0.97, range 0.55–4.58) (p*<*0.001) (Figure).

**Figure:**
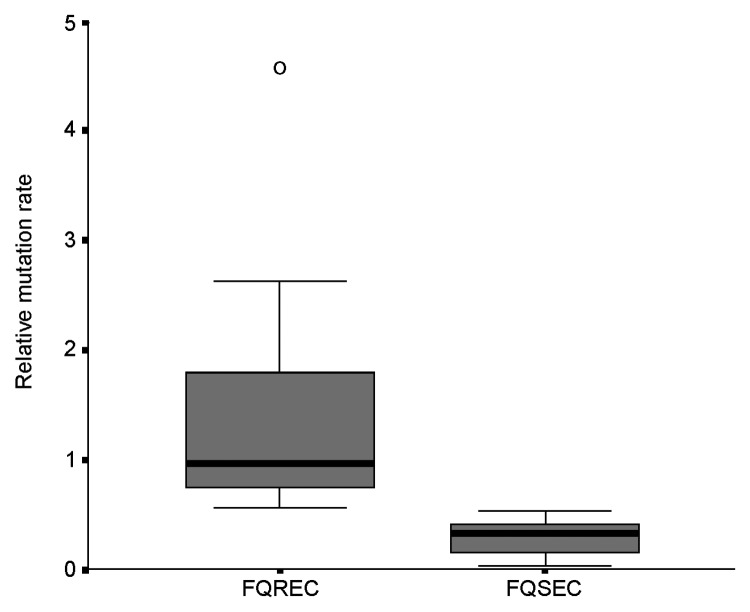
Box plot of relative mutation rate of 10 fluoroquinolone (FQ)-resistant (FQREC) and 10 FQ-sensitive (FQSEC) *Escherichia coli*.

## Discussion

In this study, we investigated the epidemiology and virulence characteristics of FQ-resistant *E. coli* collected during a large, population-based survey of ≈4,000 people in 2 cities in Indonesia (Surabaya and Semarang). The overall prevalence of resistance to ciprofloxacin was 8%, but in the fecal flora of patients at time of discharge from the hospital the prevalence was 23%.

### Dissemination of FQ-resistant *E. coli* and Mutation Rate

Three possible explanations for the high prevalence of FQ-resistant *E. coli* among patients that had been hospitalized for >5 days must be considered: transferable resistance, clonal spread, and mutation-based selection of resistance fostered by the use of antimicrobial agents. Transferable plasmid-mediated quinolone resistance has been described recently in *E. coli* from China (*17*). Wang et al. found that 6 (8%) of 78 ciprofloxacin-resistant *E. coli* strains from a hospital in Shanghai contained *qnr*. However, from the present study we cannot draw any conclusion about the contribution of this mechanism in Indonesia. As for clonal spread, molecular typing showed extensive genetic diversity among FQ-resistant isolates in Indonesia. We identified a few distinct multiple-isolate clones in the hospital environment. Although all these clonal strains were shown to be non-ExPEC, they may still pose a health threat, especially to immunocompromised patients in hospital settings. Nosocomial outbreaks of infections caused by disseminating FQ-resistant clones have already been described (*8*). However, in our study, limited clonality among isolates was found, which suggests that other factors contribute more to the high prevalence of FQ-resistant *E. coli* among hospitalized patients.

To determine whether mutation-based resistance fostered by selection pressure contributed to the prevalence of FQ-resistant *E. coli* in Indonesia, we performed a mutation rate analysis of selected isolates. We found a strong correlation between resistance and an elevated mutation rate. This finding agrees with a recent report from Komp Lindgren et al., in which high mutation rates of *E. coli* strains from urinary tract infections were strongly associated with FQ resistance (*9*). To demonstrate that this mutation-based resistance was selected for by the use of FQs, we must know the consumption figures of the quinolones. In other reports, evidence suggests that the use (and misuse) of ciprofloxacin in human and animal medicine may predispose to an increase in infections with resistant *E. coli* (*8*). As information on the use of FQs in Indonesia is currently not available, we cannot draw any conclusions on a potential link between antimicrobial drug use, selection pressure, and mutation-based resistance. Thus, based on the large clonal diversity of the FQ-resistant *E. coli* and the resistant isolates that have a slightly elevated mutation rate relative to FQ-sensitive isolates, independent emergence of new resistant mutants likely occurs regularly in this setting.

### Phylogenetic Typing and Virulence Profiling

Phylogenetic typing and virulence profiling were performed to investigate whether a potential clinical hazard was associated with the presence of these isolates. Our data on the distribution of phylogenetic groups among the 396 *E. coli* isolates are consistent with those of most other studies. In an examination of human commensal *E. coli* strains, the frequencies of B2 strains were found to be 1 (2%) of 55 in Mali, 6 (11%) of 56 in France, and 11 (19%) of 57 in Croatia (*18*). In our study, 4% of the isolates overall were of B2 origin. However, the results from a report by Zhang et al. do not agree with our data (*19*). B2 strains accounted for 42 (48%) of 88 commensal rectal strains from healthy college-aged women in Michigan. Likewise, Sannes et al. noted a high prevalence of group B2 among rectal isolates from hospitalized, elderly, male veterans in Minnesota (*20*). Differences may be due to geographic variation, differences in host population characteristics, or differences in strain characteristics such as antimicrobial resistance.

We did not observe a significant shift toward low-virulence phylogenetic groups for resistant isolates, as was reported by Johnson et al. (*10*). However, we confirmed that the isolates were notably depleted for phylogenetic group B2 and enriched for group D. We also confirmed that FQ-resistant *E. coli* exhibited sparser virulence profiles. The most prevalent VF was *iutA*, which was detected in 36% of the resistant isolates; however, this VF is less common in virulent group B2 strains (*1*). Accordingly, only 2% of the resistant isolates were found to be ExPEC. These 4 isolates also lacked the VFs *iha*, *sat*, *fyuA,* and *malX* as compared to the FQ-susceptible ExPEC. Whether ExPEC strains cause infection in humans depends on several other factors, including susceptibility of the host. Therefore, that many (36%) of the 44 ExPEC isolates were from group 2 patients who had been hospitalized for >5 days is of concern. When patients become colonized with FQ-resistant ExPEC strains in the hospital, they presumably will have an increased risk of acquiring a nosocomial infection and, when discharged with such a strain, also for community-acquired infection; in such case, an optimal therapy will be more difficult to select. Of note, a relationship has recently been shown to exist between ciprofloxacin-resistance in *E. coli* and the production of extended-spectrum β-lactamases, which would further limit therapeutic options (*21*).

Our observations provide insight into the epidemiology and virulence characteristics of FQ-resistant *E. coli* from stools of patients and healthy participants in Indonesia. The high prevalence of FQ-resistant *E. coli* in the hospital environment seems to be primarily due to a combination of limited clonal spread and the spontaneous emergence of resistant strains, possibly fostered by selection pressure. Transferable resistance, however, cannot be ruled out as an additional explanation in the present study and will be the subject of future investigations. Although the resistant isolates mainly belong to phylogenetic groups A and B1 and show a low virulence profile, similar strains have caused disease in humans (*3**,**10*). The data support the need to implement strict infection control measures in hospitals and to promote and monitor the prudent use of antimicrobial drugs. Continued surveillance of the changes of resistance patterns and virulence profiles of clinical and nonclinical *E. coli* isolates is warranted.

## Footnotes

3 ExPEC: *papEF* (P fimbrial tip pilins); *papG* (P adhesin); *papG* alleles I, II, and III; *sfaS* (S fimbriae); *focG* (F1C fimbriae); *iha* (putative adhesin-siderophore); *bmaE* (M fimbriae); *gafD* (G fimbriae); *F17a* (F17a fimbriae); *clpG* (CS31A adhesin); *afaE8* (afimbrial adhesin VIII); *fimH* (type 1 fimbriae); *cnf1* (cytotoxic necrotizing factor); *cdtB* (cytolethal distending toxin); *ireA* (siderophore receptor); *sat* (secreted autotransporter toxin); *astA* (S-like enterotoxin); *iroN* (siderophore receptor); *fyuA* (yersiniabactin receptor); *kpsM II*, K1, and K2 (*kpsM* II variants; group 2 capsule); *kpsM* III (group 3 capsule); *rfc* (O4 lipopolysaccharide synthesis); *cvaC* (colicin V); *traT* (serum-resistance associated outer membrane protein); *ibeA* (invasion of brain endothelium); *ompT* (outer membrane protease T); *iss* (increased serum survival); *usp* (uropathogenic specific protein); *malX* (marker for pathogenicity-associated island from strain CFT073); and H7 *fliC* variant (flagellin).
